# Central domain deletions affect the SAXS solution structure and function of Yeast Hsp40 proteins Sis1 and Ydj1

**DOI:** 10.1186/1472-6807-11-40

**Published:** 2011-10-19

**Authors:** Julio C Silva, Julio C Borges, Douglas M Cyr, Carlos HI Ramos, Iris L Torriani

**Affiliations:** 1 Department of Condensed Matter Physics, "Gleb Wataghin" Physics Institute, State University of Campinas (UNICAMP), Campinas, SP 13083-859, Brazil; 2 Brazilian Biosciences National Laboratory, Center for Research in Energy and Materials (CNPEM), Campinas, SP 13083-970, Brazil; 3 Institute of Chemistry of São Carlos, University of São Paulo, São Carlos, SP 13.560-970, Brazil; 4 Department of Cell and Developmental Biology, University of North Carolina, Chapel Hill, NC 27599, USA; 5 Department of Organic Chemistry, Institute of Chemistry, University of Campinas UNICAMP, SP 13083-970, Brazil; 6 European Synchrotron Radiation Facility, Grenoble, France

## Abstract

**Background:**

Ydj1 and Sis1 are structurally and functionally distinct Hsp40 proteins of the yeast cytosol. S*is1 *is an essential gene whereas the *ydj1 *gene is essential for growth at elevated temperatures and cannot complement *sis1 *gene deletion. Truncated polypeptides capable of complementing the *sis1 *gene deletion comprise the J-domain of either Sis1 or Ydj1 connected to the G/F region of Sis1 (but not Ydj1). Sis1 mutants in which the G/F was deleted but G/M maintained were capable of complementing the *sis1 *gene deletion.

**Results:**

To investigate the relevance of central domains on the structure and function of Ydj1 and Sis1 we prepared Sis1 constructs deleting specific domains. The mutants had decreased affinity for heated luciferase but were equally capable of stimulating ATPase activity of Hsp70. Detailed low resolution structures were obtained and the overall flexibility of Hsp40 and its mutants were assessed using SAXS methods. Deletion of either the G/M or the G/M plus CTDI domains had little impact on the quaternary structure of Sis1 analyzed by the SAXS technique. However, deletion of the ZFLR-CTDI changed the relative position of the J-domains in Ydj1 in such a way that they ended up resembling that of Sis1. The results revealed that the G/F and G/M regions are not the only flexible domains. All model structures exhibit a common clamp-like conformation.

**Conclusions:**

Our results suggest that the central domains, previously appointed as important features for substrate binding, are also relevant keeping the J-domains in their specific relative positions. The clamp-like architecture observed seems also to be favorable to the interactions of Hsp40 with Hsp70.

## Background

Molecular chaperones are proteins that are involved in assisting the folding and assembly of newly synthesized proteins recognizing non-native substrate proteins predominantly via their exposed hydrophobic residues [1]. However, the conditions for the successful folding *in vivo *are not always favorable. The cellular environment is crowded and thus protein denaturation and aggregation will be major problems. Thus, there is the need for chaperones that also protect cells from elevated temperature or other cellular stress situations, to achieve successful folding of proteins *in vivo*. There are several families of Heat Shock Proteins (HSPs), each family acts to assist protein folding in a different way.

An important chaperone family is the 40-kDa Heat shock protein (Hsp40). Chaperones from the Hsp40/DnaJ family play important roles in cells by working together with molecular chaperone Hsp70 to promote protein folding, assembly, translocation and degradation [2-5]. Hsp40 proteins can interact with the hydrophobic side-chains of non-native polypeptides preventing aggregation [6,7]. Hsp40 can then form transient complexes with Hsp70 presenting non-native polypeptides for subsequent protein folding [8-10]. The members of the Hsp40 family typically contain a J-domain, which regulates the ATP-dependent binding of peptides by Hsp70 [4,11,12].

**Figure 1:**
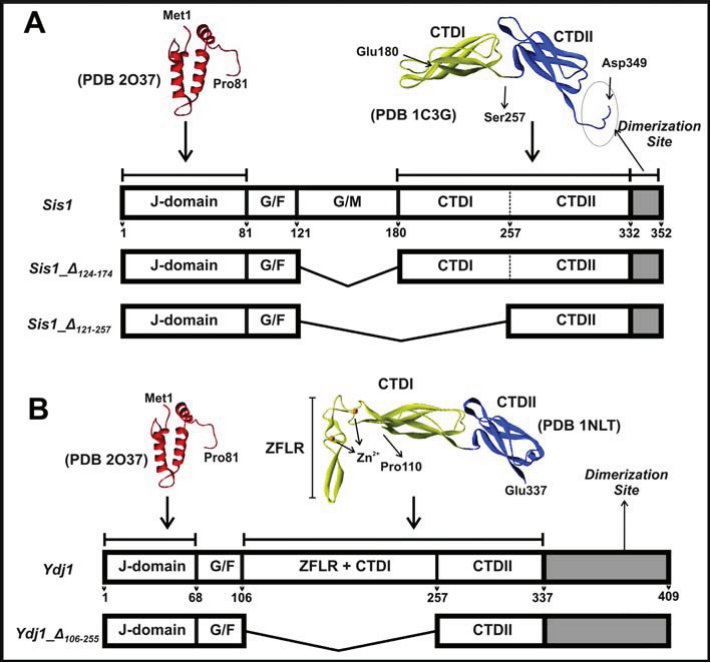
**Domain organization of the Hsp40s and mutants**. (A) Schematic representation of *Sis1 *domains from known high-resolution structures. (B) Schematic representation of *Ydj1 *domains from high-resolution structures.

Members of the Hsp40 family act as molecular chaperones to bind and deliver non-native proteins to Hsp70 and can be divided into three main groups, from which two: Type I and Type II are the most studied. The two types are not functionally equivalent [13-15] and exhibit major differences in chaperone activity [16]. In both types the J-domains are connected to the central and C-terminal domains via a G/F-rich (Glycine/phenylalanine-rich) linker (Figure 1).

Type I Hsp40s, such as *Escherichia coli *DnaJ, yeast Ydj1 and human Hdj2, contain a zinc-finger-like linker region (ZFLR) between the G/F domain and the C-terminus (Figure 1B), and Type II Hsp40 proteins such as yeast Sis1 and human Hdj1 contain a G/M-rich (Glycine/Methionine-rich) domain in the linker region (Figure 1A) [14-17]. Both types have a substrate-binding site located at their C-terminal domain, which is divided in subdomains I (CTDI) and II (CTDII) (Figure 1).

The reason why Type I and Type II Hsp40s exhibit differences in chaperone activity is unknown. Some biochemical and structural studies have already provided some insight into this question, suggesting that the answer resides in their structural differences [15,18,19]. Hence, the need for a comprehensive study of their structure is of considerable importance.

High-resolution structural studies with fragments of Sis1 and other Type II Hsp40s indicated that these proteins function as homodimers that have a clamp-like architecture and use a shallow groove located on the surface of monomers to bind non-native proteins [20,21]. In addition, the G/F-rich regions of Ydj1 and Sis1 lie adjacent to their putative polypeptide binding domains and they seem to specify the functions of these Hsp40s [22-24]. These structure/function studies together with hydrodynamic analysis showed that both types of Hsp40 proteins form dimers in solution [6,18,19]. Dimer formation plays a critical role in Hsp40s chaperone activity because disruption of the dimerization motifs results in severe defects in both chaperone functions [20,25].

Previous results showed that human and yeast Type I and Type II Hsp40s, have distinct quaternary structure [18,19]. These results raised the hypothesis that the central domains controlled the quaternary structure of both types of Hsp40s, because in chimeric mutants, in which the central domains of Ydj1 (ZFLR) and Sis1 (G/M) were switched, their properties were exchanged. A chimeric Ydj1 in which the ZFLR had been switched by the G/M from Sis1 proved to be functionally and structurally similar to Type II Sis1. Correspondingly, a chimeric Sis1 in which the G/M had been switched by the ZFLR from Ydj1 proved to be functionally and structurally similar to Type I Ydj1 [15,19].

To increase our knowledge on the role of the central domains in the structure/function relationship we defined the biophysical and functional features of Sis1 and Ydj1 mutants with deleted central domains that were specific for Type I or Type II Hsp40s. These studies entailed a functional analysis of mutated Hsp40s coupled with biophysical investigation of the quaternary structure by dynamic light scattering, analytical ultracentrifugation and small-angle X-ray scattering (SAXS). A refined solution structure of Sis1 was also obtained using improved SAXS data. The outcome of these studies showed that deletion of either the G/M or the G/M plus CTDI domain regions had minor impact on the overall quaternary structure of Sis1. Consequently, our results suggest that the central domains are important for substrate binding and maintenance of the J-domains in their specific relative positions.

## Results and Discussion

### Protein samples

**Figure 2:**
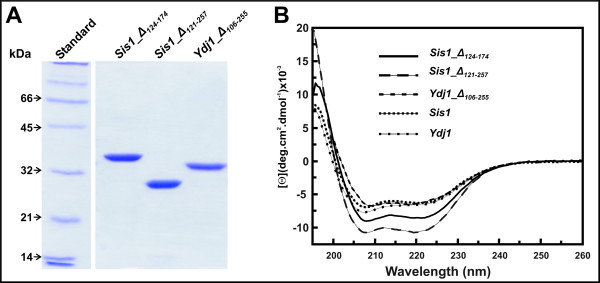
**Results from protein expression**: (A) SDS-PAGE 12% showing that the recombinant mutant proteins were > 95% pure. Molecular masses for standard proteins are shown on left. (B) CD spectra of recombinant proteins (10-40 μM) were measured from 195 to 260 nm in 25 mM Tris-HCl (pH 7.5) containing 500 mM NaCl and are shown as molar residual ellipticity ([Θ]).

Sis1 is a yeast member of the Type II Hsp40s family and contains 352 residues arranged in a highly conserved α-helical N-terminal J-domain, a disordered middle region (divided into glycine/phenylalanine (G/F) and glycine/methionine (G/M) rich regions) and two C-terminal sub-domains (CTDI and CTDII) as shown in Figure 1A. In order to understand the role of the central regions in the structure and function of Type II Hsp40s, two Sis1 deletion mutants were produced: Sis1_Δ_124-174_, from which the G/M region had been deleted, and Sis1_Δ_121-257_, from which both the G/M and the CTDI had been deleted (Figure 1A). Ydj1 is a yeast Type I Hsp40 that contains 409 residues also arranged in a highly conserved α-helical N-terminal J-domain, a disordered middle region (a glycine/phenylalanine (G/F)), a Zinc finger domain (ZFLR or Cys-rich domain) and a two C-terminal sub-domains (CTDI and CTDII) as shown in Figure 1B. Again, to understand the role of those regions in that protein structure, we also studied a Ydj1 deletion mutant that we named Ydj1_Δ_106-255_, which both the Zinc finger-like region (ZFLR) and the CTDI had been deleted (Figure 1B). The mutants were purified with no apparent contamination (>95% pure; Figure 2A) and maintained at 4°C to avoid degradation. The folded conformation of the proteins was investigated by circular dichroism (CD) spectroscopy (Figure 2B). As previously shown for the wild-type proteins Sis1 and Ydj1 [19], the mutants had CD spectra of well folded proteins with minima at about 208 and 220 nm and a positive peak bellow 200 nm (Figure 2B). The shapes of the spectra indicated that no large unfolded portion was present. This is in good agreement with the results from hydrodynamic measurements (see below).

### Hsp40 function

**Figure 3:**
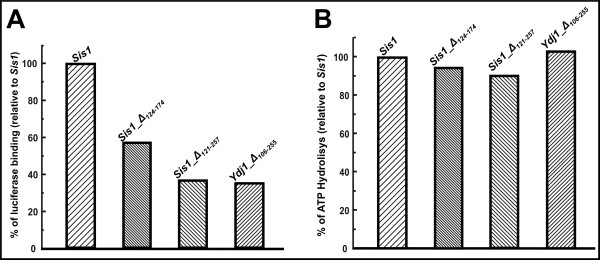
**Functional tests**: (A) Hsp40s ability to bind client proteins was about 40%. (B) Hsp40s ability to stimulate the ATPase activity of Hsp70 was about 90% compared to Sis1. (See text for more details).

Hsp40s act by binding an unfolded or partially unfolded protein (client protein) delivering it to Hsp70 and concomitantly stimulating the Hsp70 ATPase activity. ATP hydrolysis by Hsp70 is a crucial step in protein folding assisted by this chaperone. The effect of the deletions on the function of the Hsp40s was assayed by testing both the ability to bind a client protein and the stimulatory effect on the ATPase activity of Hsp70 (Figure 3). First, the ability to bind heated luciferase (a client protein) was tested and the Sis1 binding was set as standard (100%). Compared to Sis1, the efficacy of Sis1_Δ_124-174_, was of about 60%, and both Sis1_Δ_121-257 _and Ydj1_Δ_106-255 _were of about 40% (Figure 3A). Second, the mutants were assayed regarding their ability to stimulate ATP hydrolysis of Ssa1 (Hsp70) and the Sis1 stimulatory effect was set as standard (100%). Sis1_Δ_124-174 _and Ydj1_Δ_106-255 _had effect similar to that of Sis1, inside the error, and the effect of Sis1_Δ_121-257 _was of about 90% (Figure 3B). For comparison, the performance of Ydj1 in both experiments was similar to that of Sis1, within experimental error (data not shown). The results show that while the deletions decreased in about 50% the ability to bind client proteins, they seem to have no effect on the ability of the J-domain in interacting with Hsp70. These results suggested that the J-domains maintained their proper conformation and functionality.

### Hydrodynamics

**Figure 4:**
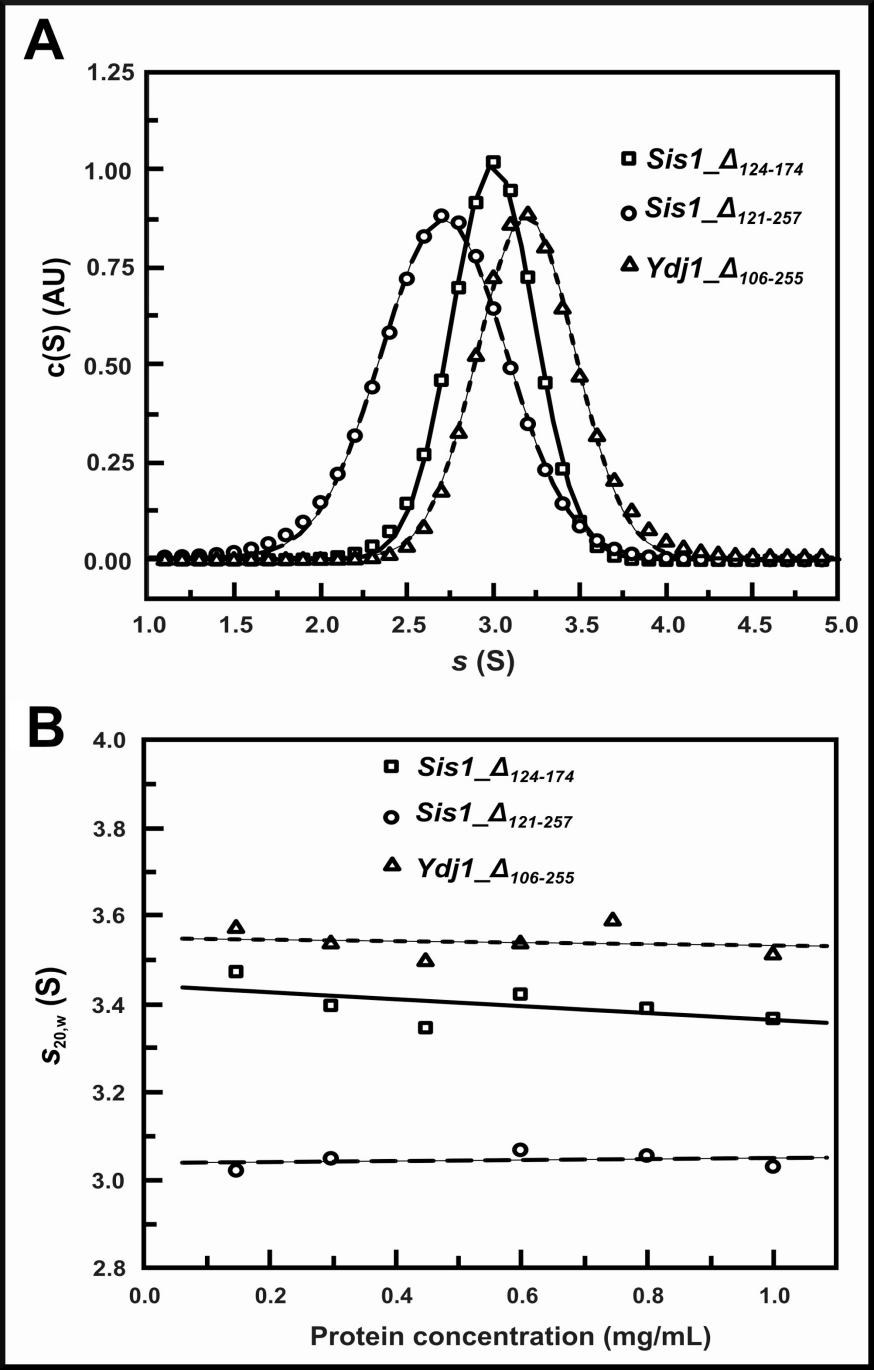
**Results from analytical ultracentrifugation (AUC) sedimentation velocity (SV) experiments**. (A) The Figure shows experiments using 1,000 μg/mL of protein concentration in 25 mmol/L Tris-HCl (pH 7.5) containing 500 mmol/L NaCl. (B) The software Sednterp was used to correct the apparent s to the s_20,w_. Proteins s^0^_20,w _values were calculated from the plot of s_20,w _versus protein concentration and are shown in Table 1.

Sis1 and Ydj1 are dimers in solution and here we used analytical ultracentrifugation (AUC) to investigate the oligomeric status of the deleted mutants. We performed AUC sedimentation velocity (SV) experiments and fitted the data using SedFit that supplied continuous sedimentation distribution c(S) (Figure 4A). From the maximum of the peaks of the c(S) curve, the apparent s, which was corrected to standard conditions (s_20,w_) and plotted against protein concentration (Figure 4B). The extrapolation of s_20,w _to 0 mg/mL gave s^0^_20,w _which is an intrinsic property of the protein and contains information about both the molecular mass (M) and the asymmetry of the molecule. Normally, a variation in the value of s^0^_20,w _induced by external factors (pH changes, salt strength, ligands or temperature) is related to conformational changes [26]. The values of s^0^_20,w _and D^0^_20,w_, obtained from dynamic light scattering (DLS) experiments, are shown on Table 1. The M values obtained from both the c(M) distribution and the s/D ratio (Equation 4), and the weight average factor ƒ/ƒ_0 _are also shown (Table 1). Our results suggested that Sis1_Δ_124-174_, Sis1_Δ_121-257 _and Ydj1_Δ_106-255 _are also dimers in solution and they have an asymmetric or elongated shape as shown previously for Hsp40 proteins [18,19].

**Table 1:** Summary of the hydrodynamic data obtained from DLS and AUC experiments.

Hydrodynamic properties	Protein			
	
	Sis1	Sis1_Δ_124-174_	Sis1_Δ_121-257_	Ydj1_Δ_106-255_
Predicted *M *(kDa)*	75.2	66.1	46.8	56.1
Experimental *M *(kDa)	64 ± 3^€^	58 ± 2^# ^58 ± 1^€^	45 ± 2^# ^43 ± 1^€^	54 ± 1^# ^55 ± 2^€^
*s*^0^_20,w _(S)^#^	3.5 ± 0.1^%^	3.4 ± 0.1	3.0 ± 0.1	3.6 ± 0.1
*s_sph _*(S)	6.5	5.9	4.7	5.2
*D*_20,w _(10^-7^cm^2^/seg)	5.1 ± 0.2	5.4 ± 0.1	6.3 ± 0.1	5.9 ± 0.2
*D*_sph _(10^-7^cm^2^/seg)	7.7	8.0	9.0	8.45
*ƒ/ƒ*_0_^#^	1.6 ± 0.1	1.6 ± 0.1	1.5 ± 0.1	1.4 ± 0.1

* *M *predicted from amino acids sequence for all proteins as dimers.

^# ^data from SV experiments fitted by SedFit software (see Material and methods for details);

^€ ^*M *calculated from *s*/*D *ratio as showed by Equation 4;

^% ^data from [19].

### SAXS results

**Figure 5:**
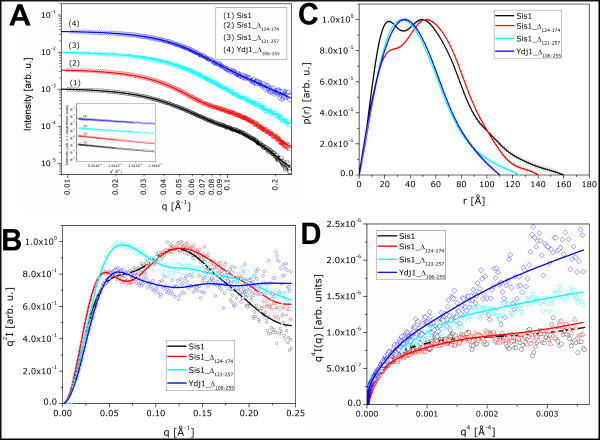
**SAXS results for the proteins Sis1, Sis1_Δ_124-174_, Sis1_Δ_121-257_, and Ydj1_Δ_106-255_**. (A) Scattering intensity curves for all the proteins. The inset shows the Guinier region showing the linear regression fit of the experimental data for all proteins. (B) Corresponding p(r) functions. (C) Kratky plots showing different degrees of flexibility for each protein. (D) Porod plots for the four proteins. A small plateau region in the curves of Sis1 and Sis1_Δ_124-174 _indicate a more defined molecular volume for those macromolecules.

To explore the impact of selective central domains deletion on Hsp40s quaternary structure, SAXS analysis was performed. The corrected and normalized experimental SAXS curves for the proteins Sis1, Sis1_Δ_124-174_, Sis1_Δ_121-257_, and Ydj1_Δ_106-255 _in the q range 0.01 < q < 0.25Å^-1 ^are displayed in Figure 5(A), together with the respective regularization fitting (solid lines) resulting from the p(r) function calculation using GNOM. The corresponding p(r) functions, shown in Figure 5(B), indicated a slightly elongated shape for all the proteins in solution, confirming the AUC results. The Kratky plots presented in Figure 5(C) showed that those proteins are all quite flexible in solution, which gave us a clue about the difficulty in crystallizing them. An inspection of the Porod plots in Figure 5(D) shows that the curves do not contain a significant plateau region indicating well defined particle volumes, but the plots for Sis1 and Sis1_Δ_124-174 _present a fairly flat region in the q^4^-range 0.0012<q^4^<0.0025Å^-4^, suggesting a more compact conformation for those two proteins. The complete deviation from the Porod regime leads to predict more flexible structures for the Sis1_Δ_121-257_, and Ydj1_Δ_106-255 _proteins. The degree of flexibility of each individual protein is thus confirmed from these plots. The inset in Figure 5(A) displays the ln(I(q)) vs. q^2 ^plot within the validity region for the Guinier approximation (qR_g_<1) together with the corresponding linear regression for each protein. The linearity of those plots confirmed the monodispersity of the samples. The maximum dimension (D_max_) values, the radii of gyration (Rg) obtained by Guinier approximation and from the p(r) functions, as well as the calculated and estimated M plus the oligomerization states of all the proteins are presented in Table 2. The Rg values obtained by the two approaches are in close agreement. The most reliable Rg value for each molecule is that obtained from the p(r) function, derived from the complete experimental curve. As described in Material and Methods, a bovine serum albumin (BSA) solution was used as reference sample for the M estimation using the SAXS data. The results indicated that all the proteins exist in a dimeric state in solution, since the values obtained are approximately twice the values calculated from the primary sequence (Table 2).

**Table 2:** Overall parameters derived from the SAXS results for the proteins Sis1, Sis1_Δ_124-174_, Sis1_Δ_121-257 _and Ydj1_Δ_106-255_

	Protein			
	
Structural properties	Sis1	Sis1_Δ_124-174_	Sis1_Δ_121-257_	Ydj1_Δ_106-255_
C (mg/mL)	7.1, 3.1	6.8, 4.2	5.2, 3.9	5.5, 2.75
Rg (Å) (Guinier)	42 ± 1	42 ± 1	35 ± 1	33 ± 1
Rg (Å) (from p(r))	43.3 ± 0.5	42.6 ± 0.3	35.2 ± 0.3	34.2 ± 0.3
Dmax (Å)	160	140	125	110
M (kDa) (SAXS)	~71	~69	~42	~47
Predicted *M *(kDa)*	75.2	66.1	46.8	56.1
Oligomerization state	Dimer	Dimer	Dimer	Dimer

* *M *predicted from amino acids sequence for all proteins as dimers.

### Ab initio and rigid body model calculations based on SAXS data

Two *ab-initio *computational routines were used to calculate the low resolution models for the molecular envelopes of the proteins Sis1, Sis1_Δ_124-174_, Sis1_Δ_121-257_, and Ydj1_Δ_106-255 _proteins. The Dummy Atoms (DA) and Dummy Residues (DR) models were derived from the X-ray scattering data introducing a 2-point symmetry constraint in the calculations. This assumption was based on the solution scattering data, which indicated that all the molecules under study were dimers in solution. The crystallographic structure that identified the Sis1 peptide binding fragment as a homodimer in the crystal [20] as well as information on the modeled dimer structure of the Ydj1 peptide binding fragment [25] were taken into account in our calculations. The q-range used in the DAMMIN model calculation was the one generally chosen for the application of this program (q_max_~ 8/R_g_). The full q-range range (0.01<q<0. 25 Å^-^1) was used for the DR and rigid body modeling routines (see Methods). Keeping in mind that SAXS *ab initio *modeling routines do not produce a unique solution, ten independent runs were performed for each calculation.

The resolution of the DA and DR models does not permit an unambiguous determination of the spatial positions of secondary structure elements, but they portray the overall recurring extended shape of the most frequent conformations adopted by these molecules in solution. A spherical start volume was used for the DA modeling calculation in order to minimize the generation of any specific direction for the p2 symmetry introduced based on the fact that all molecules were dimers. Also, since we were dealing with flexible molecules, it was important to check if the DR and Rigid Body approaches (non initial-volume dependent) were giving similar results. Consequently, rigid body (RB) calculations were performed in an attempt to obtain information on the position of the J-domain and other domains with available crystallographic data to restore the protein structures based on SAXS data. In all cases, we tried to model the molecular envelope of the protein, performing calculations with 2-point symmetry constraints because of the proteins dimeric structure as explained above. Also, as previously mentioned, the dimerization sites of all proteins were known *a priori *from their X-ray crystallographic structure and this information was used to impose dimerization contact conditions in the RB calculations. The position of the linker between the C-terminal domains and the J-domain of each protein was known from the amino acid sequences. Ten runs were performed for each set of calculations. The multiple runs gave almost coincident results and allowed the identification of the position of the individual domains in the RB models.

**Figure 6:**
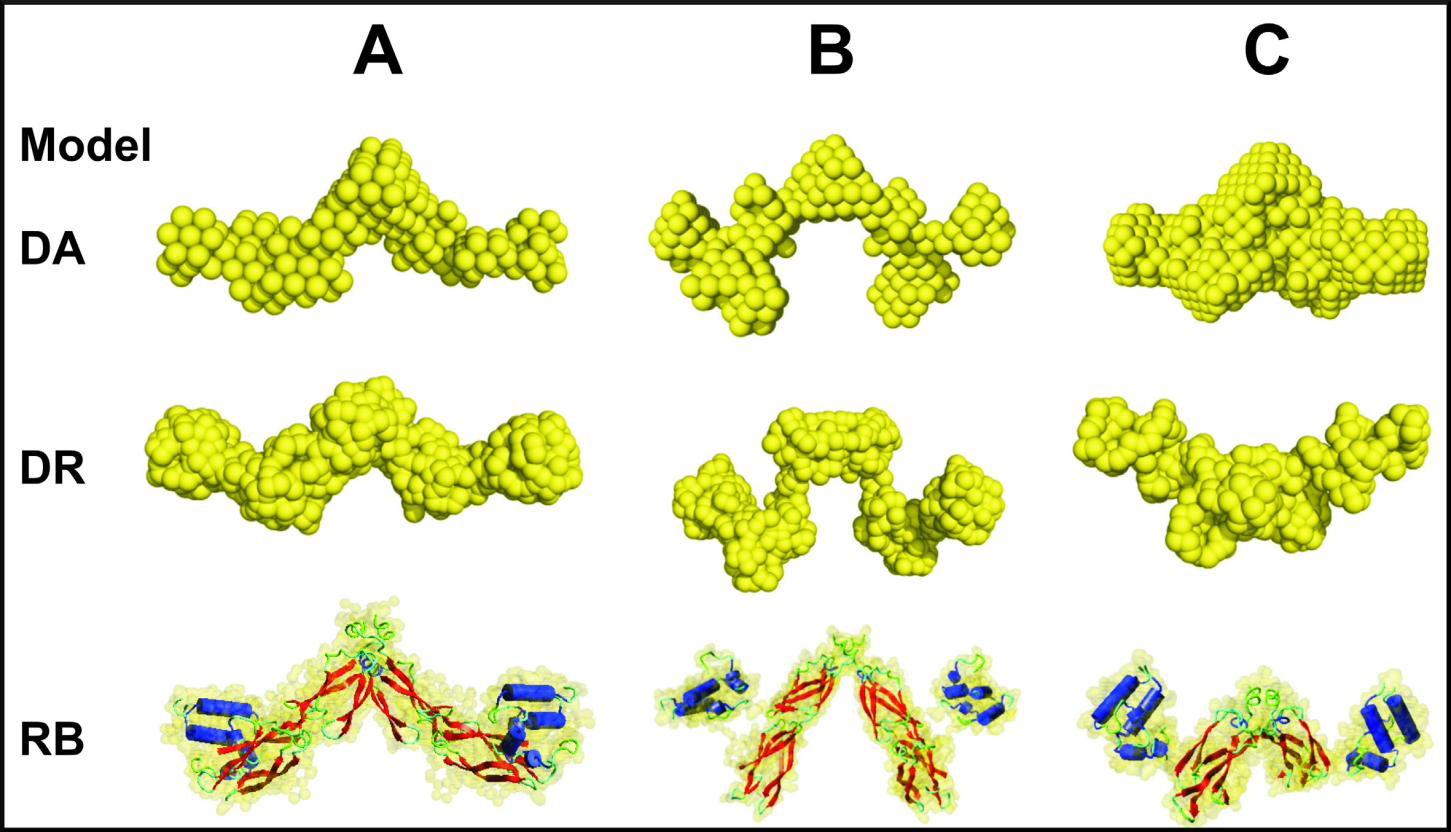
**Results of the *ab initio *(DA and DR) model calculations as well as the rigid body models are displayed for the full length Sis1 protein and its deleted mutants**. (A) Sis1, (B) Sis1_Δ_124-174_, **(C) **Sis1_Δ_121-257_.

The *ab initio *and rigid body models calculated for the full length Sis1 protein and its deleted-domain mutants are presented in Figure 6. The DA (filter averaged), DR and RB models for proteins Sis1, Sis1_Δ_124-174_, Sis1_Δ_121-257_, are shown in panels A, B and C respectively. In the case of the DR models, averaging does not add substantially to the results. So, the models displayed are those presenting the lowest normalized spatial discrepancy (NSD) values, which also showed the best agreement with the DA model. NSDs tend to zero for nearly similar objects and when they exceed 1, the objects systematically differ from one another (as explained in the Methods section). The NSDs calculated can be considered reasonably good for the DA and DR modeling approaches on account of the flexibility and conformational changes of these molecules. The itemized NSD values for each DA, DR and RB models of Sis1, Sis1_Δ_124-174_, Sis1_Δ_121-257_, are listed in Table 3.

**Table 3:** Normalized Spatial Discrepancy (NSD) range obtained by pairwise comparison of the SAXS models in each group of calculations

	Protein			
	
Modelling method	Sis1	Sis1_Δ_124-174_	Sis1_Δ_121-257_	Ydj1_Δ_106-255_
Dummy atoms	1.00 - 1.22	1.17 - 1.41	0.75 - 1.01	0.88 - 1.21
Dummy residues	1.41 - 1.97	1.84 - 2.68	1.47 - 1.76	1.38 - 1.56
*Ab initio *and rigid body	1.95 - 2.87	1.95 - 2.51	1.54 - 1.85	1.87 - 2.11

**Figure 7:**
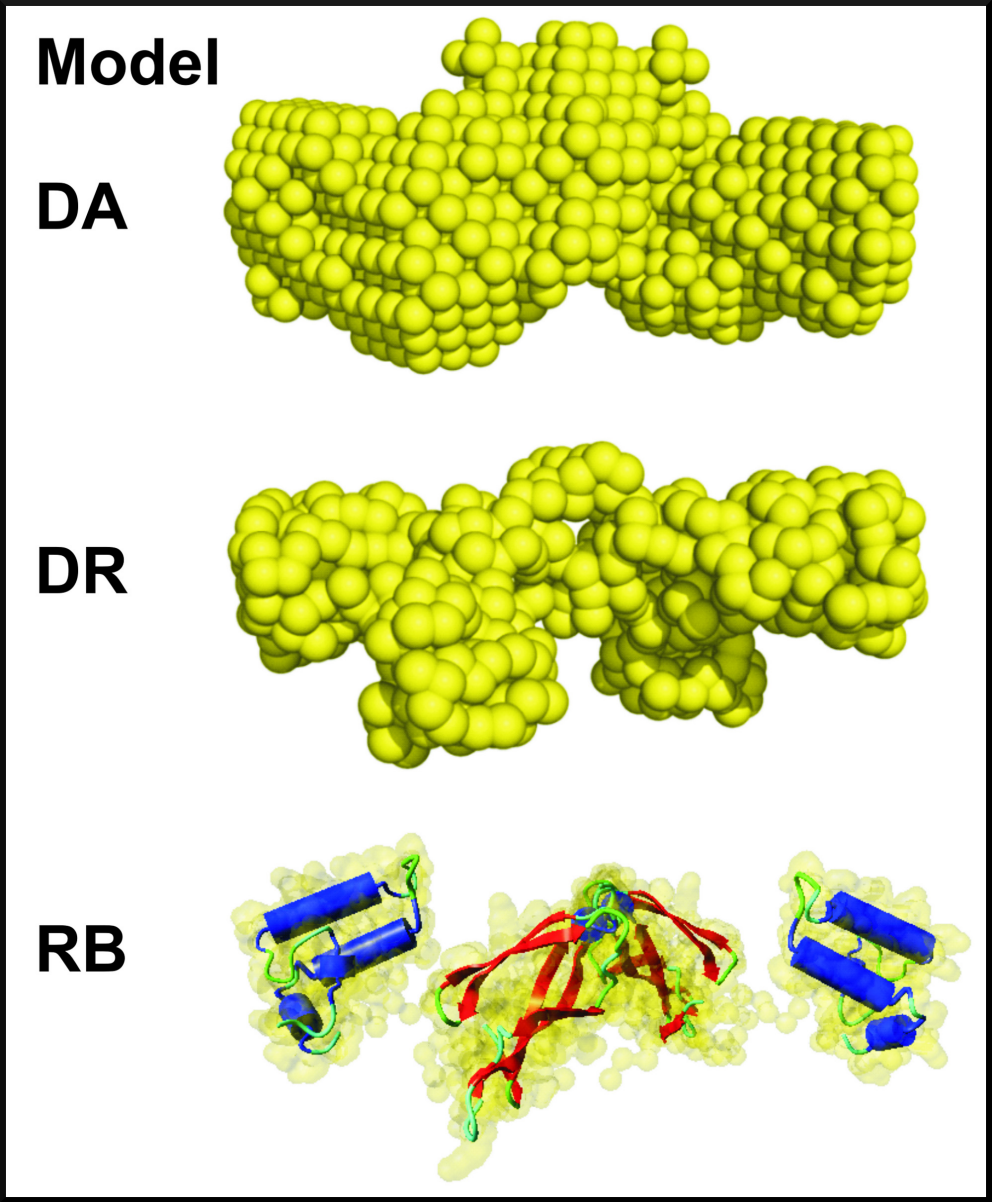
**Results of the *ab initio *(DA and DR) model calculations and the rigid body model obtained for the Ydj1_Δ_106-255 _protein, using high resolution data available for some of its domains**.

The filtered average of the *ab initio *DA model, the DR model with the lowest NSD value and the rigid body model of the Ydj1_Δ_106-255 _protein are presented in Figure 7. These results were obtained following the same procedures previously described for the Sis1 full length protein and its deleted domain mutants. The NSD values corresponding to these model calculations (Table 3) also were within the values being considered reasonably good for each different approach. The DR model with the lowest NSD value presents a remarkably good resemblance with the DA and rigid body models, and can be considered an excellent description of the Ydj1_Δ_106-255 _protein conformation in solution.

## Conclusions

In previous studies, Ydj1 and Sis1 were found to be homodimers, whose structures differed in the context of the orientation of the J-domain in relation to the long-axis of the respective proteins [18,19]. These differences in structure correlate with functional differences exhibited by Ydj1 and Sis1, and the G/F and G/M regions in these proteins were proposed to impact J-domain orientation [15]. To test this hypothesis we carried out hydrodynamic and low-resolution structural studies on Sis1 and Ydj1 deletion mutants. The deletion of the G/F region from Sis1 and Ydj1 had little impact on the function of the J-domain in regulating Hsp70 ATPase activity, but did decrease polypeptide binding activity. Surprisingly, deletion of the G/F region did not change the quaternary structure or overall flexibility of Hsp40s. However, deleting the ZFLR plus the CTDI domain in Type I Ydj1 altered the conformation of J-domains from lying along the long axis of the molecule to be orientated in a crosswise direction as in Type II Sis1. Thus, orientation of J-domains in Type I Hsp40 may be controlled by the ZFLR-CTDI, which is consistent with results from domain swap experiments [15,19]. When these domains are missing, as in the deleted mutants studied here or in the Type II Hsp40s, the J-domains become oriented in a crosswise direction. Our results also point to interactions between the ZFLR-CTDI and the J-domains as an important factor in determining the unique quaternary structure of Type I Hsp40s.

One interesting feature of Type I and Type II Hsp40s that is clear from our data is that these sub-types of Hsp40s are highly flexible. The SAXS intensity data obtained within a wider range of reciprocal space in the present experiments allowed a comparative analysis of the flexible nature of the proteins by means of the Kratky and Porod plots. These results showed that the Type I Ydj1 and Type II Sis1 Hsp40 proteins are highly flexible in solution and confirm the dimerization at the C-terminal. Besides, flexibility was observed in all constructs studied, even after deleting a flexible region (G/M). So, other flexible regions should exist in the protein in addition to G/M. Prior to the studies presented herein it was assumed that the G/F and G/M domains were the only flexible regions of Hsp40s that enabled the J-domain multiple paths to gain access to the Hsp70 ATPase domain. However, Kratky and Porod analysis of SAXS data on Sis1 and Ydj1 deletion mutants show that both remain highly flexible in the absence of the G/F region. Hsp40s bind and deliver proteins that range from nascent monomeric polypeptides to amyloid-like aggregates to Hsp70. Thus, the overall flexibility of Type I and Type II Hsp40s detected may be important to permit delivery of proteins or different sizes or assembly states to the Hsp70 polypeptide binding site and allow for simultaneous interaction of the J-domains with Hsp70s ATPase domain [5]. These results are also in agreement with crystallographic structural studies reported by Hu et al. [27], in which they observed that the CTDI of human Hsp40 may possess significant flexibility. In their work, the authors proposed an "anchoring and docking" model for Hsp40 in which the flexibility of the CTDI may be important for Hsp40 to regulate the size of the cleft in its interaction with non-native polypeptides and transfer them to Hsp70.

Functions for the G/F and G/M regions found in Type II Hsp40 in regulation of Hsp70 ATPase activity and/or substrate binding are not clear. Yet, this is an important question because a Sis1 fragment containing just the J-domain and G/F region is capable of rescuing the lethality of the *sis1*Δ strain [22]. In addition, amino acid residues in the G/M region appear to play a role in specification of Sis1 function [28]. Studies with forms of Sis1 in which the G/M domain was deleted show that loss of the G/M impairs the ability of Sis1 to bind denatured luciferase, but have no effect on regulation of Hsp70 ATPase activity. The G/M region lies adjacent to the hydrophobic grove in CTDI implicated as a polypeptide binding site and methionine has a hydrophobic side chain, which may help to build the proper binding site. Thus, the G/M region helps specify Type II Hsp40 function by assisting in substrate binding.

Since the effects on function are likely to be related to changes in the structure of the Hsp40s, one important objective of this work was to obtain low resolution models of the mutants under study using the latest computational methods available for the spatial representation of these molecules in solution using small angle scattering data. To date, there are crystallographic structures of isolated domains and the quaternary structure of Hsp40s is mainly known as a result of SAXS data from these proteins in solution, combined with other hydrodynamic techniques. Previous structural studies have also proved that Type I Ydj1 and Type II Sis1 have distinct functions and quaternary structures [15,19]. In this work, estimation of molecular masses from SAXS and AUC data indicated that the deletion mutants in solution dimerized at the C-terminal, just like the full-length protein. Thus, it is clear that the C-terminal region is very important for dimerization of both Type I and Type II Hsp40s.

Using three different low-resolution modeling methods, we obtained molecular envelopes for each protein. The rigid body modeling method seemed to be the most appropriate for flexible proteins like Hsp40 and also elucidated the domain arrangement which is important to understand possible functions of the deleted domain mutants in the cell. Interestingly, even deleting some flexible domains of Sis1 and Ydj1, the constructs kept their flexibility and maintained the clamp-like architecture of the full-length protein with the J-domains pointing outwards in opposite directions. This architecture seems to be favorable to the interactions of Hsp40 with Hsp70. The models we built for the proteins seem to agree with the anchoring and docking model, proposed by Qian et al. [10], describing how Hsp40 facilitates the delivery of non-native polypeptides to Hsp70.

## Methods

### Protein expression and purification

The recombinant protein Sis1 and Ydj1 were expressed and purified by two chromatographic steps as previously described [15,19,21]. Two Sis1 mutants were prepared from DNA constructions and expressed in *Escherichia coli *BL21(DE3)pLys: Sis1_Δ_124-174 _(pET11aSIS1_Δ_124-174_), deleting from residues 124 to 174, and Sis1_Δ_121-257 _(pET11aSIS1_Δ_121-257_), deleting from residues 121 to 257. Additionally, one DNA construction for Ydj1 mutant (pET11aYdj1_Δ_106-255_) deleted from residues 106 to 255, was prepared and was also expressed in *E. coli *BL21(DE3) strain. Cells were grown at 37°C up to an optical density at 600 nm of 0.7. The temperature was reduced to 30°C and the protein expression was induced with 0.4 mM isopropyl thio-β-D-galactoside (IPTG) during 4 hours. Thereupon, the cells were harvested by centrifugation during 10 min at 2,600 × g. The pellet was ressuspended in 50 mM Tris-HCl (pH 8.0), 500 mM KCl and 1 mM EDTA (15 mL/L of LB medium). The cells were lysed by adding 30 μg/mL of lysozyme (Sigma) and 5 U of DNAse (GIBCO BRL), kept for 30 min at ice bath, and then disrupted by sonication and centrifuged (30 min at 26,000 × g).

The purification of the proteins was performed as previously described [16,29]. Summarily, Sis1, Sis1_Δ_124-174 _and Sis1_Δ_121-257 _were submitted to a cationic chromatography in a Macro-prep^(TM) ^High S Support resin (BioRad) using an ÄKTA FPLC device (Pharmacia Biotech). The resin was equilibrated with 20 mM Tris-HCl buffer (pH 7.5) and 20 mM NaCl. Ydj1 and Ydj1_Δ_106-255 _were submitted to an anionic chromatography in a Macro-prep^(R) ^High Q Support resin (BioRad) using an ÄKTA FPLC device (Pharmacia Biotech). The proteins were eluted by NaCl gradient, dialyzed overnight against buffer 20 mM Phosphate (pH 7.5), and further purified by chromatography in a CHT^TM ^Ceramic Hydroxyapatite Type II resin (BioRad) at an ÄKTA FPLC (Pharmacia Biotech). The target proteins were eluted by a phosphate gradient. Ydj1_Δ_106-255 _was further purified by a size exclusion chromatography in a Superdex 200pg using an ÄKTA FPLC (Pharmacia Biotech) previously equilibrated with 25 mM Tris-HCl buffer (pH 7.5) and 500 mM NaCl. The efficacy of the purification was checked by 12% SDS-PAGE. Unless stated otherwise, all proteins were diluted in buffer 25 mM Tris-HCl (pH 7.5) containing 500 mM NaCl.

### Circular dichroism

Circular dichroism (CD) measurements were performed using a Jasco J-810 spectropolarimeter coupled to a Peltier-type System PFD 425S for temperature control and optimized for best performance as previously described [30]. The proteins were re-suspended in buffer 25 mM Tris-HCl (pH 7.5) containing 500 mM NaCl. Proteins concentration ranged from 10 to 40 μM and the spectra were collected at a scan rate of 50 nm/min with a spectral bandwidth of 1 nm and using a 0.2 mm path length cell.

### Chaperone activity

Hsp40s activities were tested by their ability to bind heated denaturated luciferase and to stimulate Hsp70 Ssa1 ATPase activity as previously described [15,21]. The ability to bind heated luciferase (a client protein) was tested and the Sis1 binding was set as standard (100%). With respect to Hsp40s ability to stimulate the ATPase activity of Hsp70, the mutants were assayed regarding their ability to stimulate ATP hydrolysis of Ssa1 (Hsp70) and the Sis1 stimulatory effect was set as standard (100%).

### Dynamic Light Scattering

The experimental diffusion coefficient (D) was obtained by dynamic light scattering (DLS) using a DynaPro-MS/X device (Protein Solutions). The experiments were performed at 20°C, and proteins concentration ranged from 0.5 to 2.0 mg/mL. The D value was corrected to standard conditions (D_20,w_) and extrapolated to 0 mg/mL concentration (D^0^_20,w_) in order to avoid effects of viscosity and temperature. D is related to the frictional coefficient (ƒ) by the following equation:

(1) $$D=\frac{RT}{N_{A}f}$$

where T is the absolute temperature, R is the gas constant and N_A _is the Avogadro's number.

For a protein with known Stokes radius (Rs) and viscosity (η), ƒ can be obtained applying the Stokes equation:

(2) f=6πηRs

For comparison, the frictional coefficient for a spherical particle (ƒ_0_) can be calculated using the predicted Stokes radius (R_0_) for a smooth and compact spherical protein of molecular mass M:

(3) $$R_{0}={\left(\frac{3MV_{bar}}{4\pi N_{A}}\right)}^{1∕3}$$

where V_bar _is the partial specific volume, ƒ_0 _is used to obtain the maximum diffusion coefficient (D_sph_) applying equation 1 and the frictional ratio (ƒ/ƒ_0_) is used to indicate particle asymmetry when compared to a globular protein of same M giving information on the shape of the proteins [26,31].

### Analytical Ultracentrifugation

Analytical ultracentrifugation (AUC) experiments were performed in a Beckman Optima XL-A analytical ultracentrifuge. Sedimentation velocity (SV) experiments were carried out in concentrations ranging from 150 to 1,000 μg/mL. The SV experiments were performed at 20°C, using 30,000 rpm (AN-60Ti rotor) for Sis1_Δ_121-257 _and Ydj1_Δ_106-255_, and 25,000 rpm for Sis1_Δ_124-174_. The SedFit software (Version 9.4) was used to deconvolute the sedimentation and diffusion data in order to obtain the continuous sedimentation distribution c(S) and a weight average value of frictional ratio. The ƒ/ƒ_0 _value was used as a parameter of the regularization function and also used to estimate the molecular mass from the c(M) plots [32,33]. The apparent sedimentation coefficients (s) were obtained from the maximum peak values of the c(S) curves. The standard sedimentation coefficients (s_20,w_) at each protein concentration were estimated to avoid interferences caused by viscosity and density increment [26,31]. The Sednterp software http://www.jphilo.mailway.com/download.htm was used to estimate important hydrodynamic parameters: (1) the partial specific volume (V_bar_) for Sis1 (0.7263 mL/g), Sis1_Δ_124-174 _(0.7312 mL/g), Sis1_Δ_121-257 _(0.7284 mL/g) and Ydj1_Δ_106-255 _(0.7331 mL/g) from their amino acid sequence; (2) the s_sph _and D_sph _for a globular protein of same molecular mass M and buffer viscosity (η = 1.0605 x10^-2 ^poise) and density (ρ = 1.01938 g/mL); and (3) to correct the apparent value of s to s_20,w_. The standard sedimentation coefficient extrapolated to 0 mg/mL (s^0^_20,w_) was calculated by linear regression from values of s_20,w _versus the protein concentration. The molecular mass values were obtained as the ratio of the sedimentation to diffusion coefficient using the following equation.

(4) $$M=\frac{sRT}{D\left(1-V_{bar}\rho \right)}$$

### Small-Angle X-ray scattering experiments

SAXS experiments were performed at the D02A-SAXS2 beamline of the Laboratório Nacional de Luz Síncrotron (LNLS, Campinas-SP, Brazil). The X-ray scattering data were recorded using a two-dimensional position-sensitive MARCCD detector. The measurements were performed with a monochromatic X-ray beam (wavelength of λ = 1.488 Å) and a sample-to-detector distance of 1374.4 mm, corresponding to the scattering vector range of 0.01 < q < 0.25Å^-1^, where q is the magnitude of the q-vector defined by (2θ is the scattering angle). The samples were placed in a 1-mm path length cell with mica windows [34]. The scattering patterns were recorded at two different sample concentrations for each sample: 7.1 and 3.1 mg/mL for Sis1, 6.8 and 4.2 mg/mL for Sis1_Δ_124-174_, 5.2 and 3.9 mg/mL for Sis1_Δ_121-257_, and 5.5 and 2.75 mg/mL for Ydj1_Δ_106-255_. All the samples were measured in buffer 25 mM Tris-HCl (pH 7.5) containing 500 mM NaCl. Three successive frames of 300s each were recorded for each sample and two frames more for the buffer. The scattering curves were individually corrected for detector response and scaled by the incident beam intensity and the samples absorption. The corrected buffer scattering curve was subtracted from the corresponding sample scattering. The resulting curves were normalized by the respective concentrations and carefully inspected to check for possible radiation-induced damage and concentration effects, but such effects were not observed. A 5.6 mg/mL bovine serum albumin (BSA, 66 kDa) solution was used as a standard sample to determine the molecular mass of the proteins. The molecular mass was estimated by comparison of the extrapolated value of the intensity at the origin value, I(0), of the samples scattering data with that from the reference solution of Bovine Serum Albumin (BSA) as described in Orthaber et al. [35] and Mylonas et al. [36].

### SAXS data analysis

Determination of the radius of gyration (R_g_) was performed using the Guinier approximation:

(5) $$I\left(q\right)=I\left(0\right)\exp\left(-q^{2}R_{g}^{2}∕3\right)$$

valid in the q-range where qR_g_<1 [37-39]. The linearity of the scattering curves in the validity region confirmed monodispersity of the samples and allowed further analysis. Moreover, R_g _values were also evaluated from the pair distance distribution function p(r) calculated from the scattering intensity data by means of the Indirect Fourier Transform package GNOM [40]. The p(r) function also provided the maximum dimension D_max _of the molecule, because p (r ≥ D_max_) = 0 [38,39]. Both the Guinier approximation, and the calculated p(r) function provided values for the forward scattering intensity I(0). These I(0) values were used for the estimation of the molecular mass. The confirmation of the monodispersity and dimerization of the Sis1, Sis1_Δ_124-174_, Sis1_Δ_121-257_, and Ydj1_Δ_106-255 _proteins was inferred from the molecular mass obtained for these proteins. The flexibility of the molecules was estimated from the scattered intensity analyzing the behavior of the Kratky curves (q^2^I(q) versus q) [38,39] and Porod plots (q^4^I(q) versus q^4^). These representations provided qualitative information about the degree of flexibility and compactness [38,39,41-43].

### Ab initio modeling based on SAXS data

Ab initio calculations based on SAXS data were performed to obtain low-resolution models for the conformation of the following proteins: Sis1, Sis1_Δ_124-174_, Sis1_Δ_121-257_, and Ydj1_Δ_106-255_. Two different ab initio approaches were applied using the dummy atoms (DA) and dummy residues (DR) modeling methods. Given the existence of a certain flexibility in these proteins those two types of calculations were necessary to identify the existence of common structural features between the different models. The dummy atoms modeling method provided a bead model whose calculated intensity fitted the experimental SAXS curve (see Figure S1 in Additional file 1). This DA approach was implemented using the program DAMMIN [44] in the q-range (q_max _<8/R_g_) which ends up being slightly different for each protein.

Since no unique solution can be obtained from this calculation, several independent calculations were performed. Thereupon, the models were pair wise compared and then averaged using programs of the DAMAVER suite [45] and SUPCOMB [46]. The latter program aligns two models represented by ensembles of points by minimizing a dissimilarity measure called Normalized Spatial Discrepancy (NSD). Generally, NSD values tend to zero for increasingly similar objects; when they significantly exceed 1, the objects systematically differ from one another [46]. Subsequently, new calculations were performed for each protein using the DR approach. The dummy residues modeling method provides further insights into the possible three dimensional conformation of the proteins and their deleted mutants in solution. This was implemented using the program GASBOR [47] using the full range of q values. Again, several independent calculations were performed and the NSD values were evaluated. However, an average of the DR representations does not substantially improve the quality of the models due to the flexibility of the molecules. So, a quantitative analysis of the NSD values obtained from the several models was also performed in order to obtain the most appropriate molecular conformation (i.e., the model having the lowest average NSD value), to describe the low resolution structure of the protein.

### SAXS-based Modeling of the multidomain arrangement for all proteins studied

The topology of each protein and each deleted-domain mutant of Sis1 was examined by applying a rigid body modeling method to the SAXS data. This approach employs a simulated annealing protocol to find the optimal positions and orientations of high-resolution structures of the known regions of the protein. At the same time, the conformation obtained for the unknown regions (flexible linker attached to the appropriate residues of the domains) was calculated finding the best fit to the experimental scattering data. The rigid body calculations were implemented by the program BUNCH [48] using the full q-range. In order to construct the model of the mutants of Sis1, we used the high resolution structure of the Sis1 C-terminal peptide-binding domain and the Sis1 J-domain, both found in the Protein Data Bank http://www.rcsb.org identified by the codes 1C3G and 2O37 respectively. To compose the model for the Ydj1 deleted domain mutant, we used the high resolution structure of the Ydj1 dimerization domain (PDB code 1XAO) and also the J-domain from the Sis1 structure (PDB code 2O37). Several independent calculations were performed for each protein. We applied a 2-point symmetry constraint to the model calculations, using the a priori knowledge that all of them were dimers in solution. The NSD values were evaluated in order to select the most typical model for each protein. The fits for the models (Figure S1) and the corresponding chi values (Table S1) can be found in additional files 1 and 2 respectively.

## Authors' contributions

ILT and CHIR conceived and designed the experiments. JCS performed the SAXS experiments, data analysis, modeling calculations and interpreted them together with ILT. JCB produced and purified all proteins, executed the hydrodynamics and spectroscopic experiments and interpreted them together with CHIR. DMC produced the deletion mutants and performed the functional experiments. All authors organized the results, wrote the manuscript, read and approved the final version of the manuscript.

## Supplementary Material

Additional file 1 **Figure S1**. Model fits for the four proteins studied in this work. Click here for file

Additional file 2 **Table S2**. Chi-values (root mean square of χ^2^) obtained from the fit of the theoretical scattering calculated from the models of the experimental intensity curve. Click here for file
