# Supplementary figures and images for: Central domain deletions affect the SAXS solution structure and function of Yeast Hsp40 proteins Sis1 and Ydj1
Source: BMC Struct Biol. 2011 Oct 19;11:40. doi: 10.1186/1472-6807-11-40 (PMC3236591; doi:10.1186/1472-6807-11-40)

Additional file 1

**
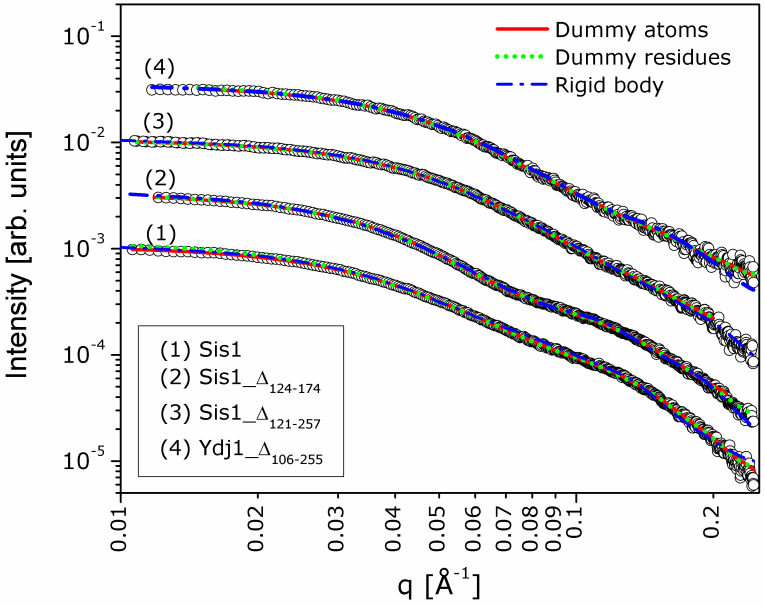
**

**Figure S1**. Model fits for the four proteins studied in this work.

Supplement: Additional file 1 — Figure S1. Model fits for the four proteins studied in this work. [file 1472-6807-11-40-S1.DOC]
