# Supplementary material for: Central domain deletions affect the SAXS solution structure and function of Yeast Hsp40 proteins Sis1 and Ydj1
Source: BMC Struct Biol. 2011 Oct 19;11:40. doi: 10.1186/1472-6807-11-40 (PMC3236591; doi:10.1186/1472-6807-11-40)
Supplement: Additional file 2 — Table S2. Chi-values (root mean square of χ2) obtained from the fit of the theoretical scattering calculated from the models of the experimental intensity curve. [file 1472-6807-11-40-S2.DOC]

Additional file 2

**Table S1** – Chi-values (root-mean square of χ^2^) obtained from the fit of the theoretical scattering calculated from the models to the experimental intensity curve.

| Modelling method | Protein | | | |
| --- | --- | --- | --- | --- |
|  | *Sis1* | *Sis1_Δ_124-174_* | *Sis1_Δ_121-257_* | *Ydj1_Δ_106-255_* |
| Dummy atoms | 0.27 – 0.36 | 0.22 – 0.23 | 0.30 – 0.31 | 0.27 – 0.40 |
| Dummy residues | 0.8 – 1.0 | 0.27 – 0.40 | 0.30 – 0.39 | 0.32 – 0.44 |
| Ab initio and rigid body | 0.8 – 1.3 | 0.4 – 0.6 | 0.34 – 0.74 | 0.6 – 1.0 |
